# Causal attributions of poverty: a social stratification analysis

**DOI:** 10.3389/fsoc.2025.1591235

**Published:** 2025-06-10

**Authors:** Lionel Marquis, Ursina Kuhn, Robin Tillmann

**Affiliations:** ^1^Institut d’Etudes Politiques, University of Lausanne, Lausanne, Switzerland; ^2^FORS (Swiss Centre of Expertise in the Social Sciences), Lausanne, Switzerland

**Keywords:** poverty, poverty attribution, social stratification, social class, panel, Switzerland

## Abstract

This study investigates the causal attributions of poverty among Swiss citizens using longitudinal data from the Swiss Household Panel (2019–2021). Agreement with the following four explanations of poverty was measured: “the poor are lazy” (individual blame), “the poor are unlucky” (individual fate), “the poor are victims of social injustice” (social blame) and “poverty is an inevitable consequence of the modern world” (social fate). Social blame shows the highest prevalence in Switzerland, followed by individual fate which has further increased over the COVID-19 pandemic. We focus on the relationship between poverty attributions and socio-economic stratification from a cross-sectional and dynamic perspective using pooled OLS and fixed effects models. The potential mechanisms discussed for individual and social blame involve self-interest, self-serving bias, socialization, exposure to poverty, resentment, and ideology. We take an encompassing view of social stratification, including education, income, wealth, deprivation, income mobility and social class. Our findings partially support the self-interest and self-serving bias mechanisms, with higher social positions correlating positively with individual blame attributions and negatively with social blame attribution. However, the socialization hypothesis is also supported, as higher education levels are associated with social blame attributions and poverty attributions do not react to changes in social stratification in the short term. Although poverty attributions vary relatively strongly within individuals over time, social stratification cannot explain intra-individual changes over time.

## Introduction

The persistence of poverty and socio-economic inequalities in contemporary societies has occupied generations of scholars, practitioners and political leaders from various backgrounds. Indeed, all major schools of thought since antiquity (whether philosophical, religious, political, or scientific) have paid some attention to the social division between rich and poor and its consequences for society. Because of their influence on the development of welfare institutions and the implementation of social policies, these “expert” judgments about the causes of poverty and inequality are extremely important (Katz, 1995, 2013; Handler and Hasenfeld, 2007; Jones, 2011). In democratic societies, however, *public opinion* plays an additional, independent role in the decision-making process. Public support for welfare institutions and social policies sets general limits to the legitimacy and acceptability of social protection systems and reforms. Therefore, “lay” opinions on the causes of poverty (as opposed to “expert” opinions) are an important source of attitudes toward welfare policies. Depending on which particular causes of poverty are considered important by citizens, their attitudes toward welfare policies will tend to reflect a “pro-welfare” or “anti-welfare” stance (Marquis and Rosset, 2021).

As public attitudes operate as “opinion dikes” constraining governments’ policy agendas (Key, 1961; Jones and Baumgartner, 2004), citizens’ explanations of poverty can have important indirect effects on social policy. For example, if these attributions are primarily “individualistic” (i.e., blaming poverty on the poor themselves), it may be easier to maintain minimal levels of social protection or to justify welfare retrenchment policies. Conversely, when poverty attributions are predominantly conceived as “structural” (i.e., blaming poverty on social causes), there may be less leeway to reform welfare systems.

Accordingly, research over the last 50 years has paid increasing attention to causal attributions for poverty in mass publics. Most studies on poverty attributions use them as explanatory factors for welfare state preferences or other variables. However, the literature has also identified many variables that *predict* inter-individual variation in poverty attributions, such as socio-economic and socio-demographic characteristics, religion, values, political attitudes, institutions or cultural characteristics at the aggregate level (for a review, see Marquis and Kuhn, 2024). With few exceptions, the empirical evidence comes from cross-sectional studies and, accordingly, could not address changes in poverty attributions over time in the population and within individuals.

In this study, we examine how social stratification is related to poverty attributions. Although some studies have examined the impact of socio-economic variables, they have considered only a few indicators and did not discuss the underlying mechanisms explicitly. In this contribution, we aim to fill this gap by taking an encompassing view of stratification, including education, current and past financial resources, deprivation, occupation and self-assessed social class and social origin. We discuss potential causal mechanisms and derive testable hypotheses. To do so, however, requires us to provide a careful description of the conceptual space underlying causal attributions of poverty.

We implement a four-category typology distinguishing between four causes of poverty: “individual blame” (**IB**: the poor are lazy), “individual fate” (**IF**: the poor are unlucky), “social blame” (**SB**: the poor are victims of social injustice) and “social fate” (**SF**: poverty is an inevitable consequence of the modern world). Unlike most previous approaches, we measure these four types of poverty attributions with separate questions asking respondents how much they agree with each explanation. These questions were introduced in the Swiss Household Panel (SHP) in the three waves from 2019 to 2021 and thus cover the period immediately before and during the Covid pandemic. In contrast to other data sources, the SHP *combines* (1) measures of poverty attributions, (2) a multifaceted measurement of social stratification variables, and (3) a longitudinal dimension allowing for the assessment of individual change in poverty attributions. The present study is, thus, an attempt to shed light on the (trans-)formation of poverty attributions and to contribute to a better understanding of their determinants.

We estimate two types of models: pooled OLS-regression and fixed effects models for time-varying independent variables. The latter analyze variance within individuals over time. To the best of our knowledge, this perspective for explaining poverty attributions has not been explored to date.

This contribution is organized as follows. In Section 2, we examine the concept of poverty attributions and present the theoretical background and measurement strategy for the four-category typology. In Section 3, we review the literature on determinants of poverty attributions related to social stratification and derive testable hypotheses—in short, who tends to attribute poverty to what type of causes, why, and in which context? Section 4 is devoted to the operationalization of our variables and to our empirical models, which are put to test in Section 5 before a concluding section.

## Causal attributions of poverty

The American sociologist Feagin (1972) is often credited with providing the first systematic evidence of the underlying structure of poverty attributions. He identified three main types of causes reported by ordinary citizens to explain poverty: (1) **individualistic** (e.g., lack of effort and willpower, lack of thrift, loose morals and drunkenness); (2) **structural** (e.g., poor education system, low wages and unemployment, prejudice and discrimination against the poor); and (3) **fatalistic** (bad luck, disability and illness, lack of ability and talent). In other words, the main drivers of poverty can be seen as either personal characteristics and behavior, the wider socioeconomic environment, or circumstances beyond the control of both the individual and the society. Another major finding of Feagin’s study was the prevalence of individualistic accounts of poverty, at least in the American context.

Following in these footsteps, many studies have reproduced the tripartite structure of poverty attributions, using factor analyses of various items (e.g., Feather, 1974; Singh and Vasudeva, 1977; Smith and Stone, 1989; Niemelä, 2008; Kreidl, 2000; Sun, 2001; Hunt, 2002, 2004; Hayati and Karami, 2005; Weiss and Gal, 2007; Habibov, 2011; Davids and Gouws, 2013; Ige and Nekhwevha, 2014; Norcia and Rissotto, 2015). However, there has been some concern about the validity of the fatalistic type, which sometimes appeared to be poorly differentiated from the structural type or internally divided into different components (e.g., Furnham, 1982a, 1982b; Payne and Furnham, 1985; Bullock, 1999; Bullock et al., 2003), leading some studies to omit the fatalistic category altogether (e.g., Kluegel and Smith, 1986; Kluegel et al., 1995; Hunt, 1996; Shirazi and Biel, 2005; Schneider and Castillo, 2015). In addition, several scholars (Cozzarelli et al., 2001; Bullock et al., 2003; Tagler and Cozzarelli, 2013) warned that the original items sets devised in the 1970s and 1980s were becoming outdated and failed to take into account the poverty consequences of new social risks (e.g., lone parenthood) and long-ignored structural problems (e.g., sexism, long-term unemployment). With the inclusion of new items, Cozzarelli et al. (2001) observed that a “subculture of poverty” category (“breakdown of the nuclear family, bad schools, being born into poverty, etc.”; p. 217) replaced the fatalistic category as the third factor to emerge from their analysis (see also Smith and Stone, 1989; Reyna and Reparaz, 2014; Bennett et al., 2016; Fung et al., 2023). Finally, it should be noted that the structure of poverty attributions depends on the questions asked in surveys and on the context. Thus, some studies have proposed a reinterpretation of the three initial factors or additional factors (e.g., Nilson, 1981; Morçöl, 1997; Bullock, 2004; Brimeyer, 2008; Niemelä, 2011; Robinson, 2011; da Costa and Dias, 2014; Mickelson and Hazlett, 2014; Homan et al., 2017). A prominent example is the explanation of poverty in poor countries (rather than domestic poverty in Western countries), for which five distinct attributional dimensions have been uncovered (e.g., Harper et al., 1990; Carr and MacLachlan, 1998; Hine and Montiel, 1999; Campbell et al., 2001; Vázquez et al., 2010).

The factor-analytic method has been used predominantly in American research. Although it is a powerful and flexible way of measuring poverty attributions in a particular context, place and time, the countless variations in questionnaires and procedures prevent systematic comparisons between studies and the drawing of useful generalizations. In contrast, much of the European research on poverty attributions over the last five decades has relied on a four-type classification of poverty attributions, which facilitates comparative analysis. Nevertheless, this advantage comes at the cost of rigidity and a shallow theoretical basis for the measures, which we discuss in more detail below.

In the four-type classification approach, which we adopt in this study, the question and response options are as follows: *Why in your opinion are there people who live in poverty? Here are four opinions: which is closest to yours?*

- because they are unlucky
- because of laziness and lack of willpower
- because of injustice in our society
- because it’s an inevitable part of modern progress.

**Figure 1:**
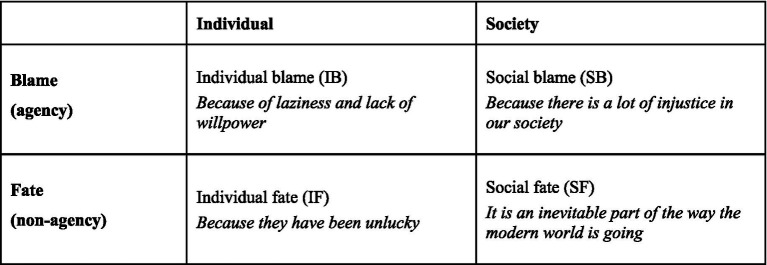
Poverty attributions according to the 4-type classification.

This four-type classification results from combining two supposedly independent dimensions of judgment about the causes of poverty (see Figure 1). The first dimension refers to the *level of explanation*—whether judgments locate the explanation for poverty at the individual level or at the social level. The second dimension refers to the notion of *agency* and distinguishes perceptions that individuals or society are responsible for poverty (“blame”) from perceptions that poverty arises from circumstances and events beyond control of individuals or social institutions (“fate”). Following van Oorschot and Halman (2000), we refer to the “unlucky” item as “**individual fate**” (IF), the “laziness” item as “**individual blame**” (IB), the “injustice” item as “**social blame**” (SB), and the “inevitable” item as “**social fate**” (SF). It is also important to note that this standard question implies a *forced choice* between one of the four response options.

Comparing this four-type typology with Feagin’s (1972) initial proposal, it is quite clear that the first three types (IF, IB, and SB) correspond to the distinction between fatalistic, individualistic and structural attributions. In contrast, the social fate type was not part of the original three-type classification. Not surprisingly, criticisms of the four-type classification of poverty attributions have focused mainly on its “forced-choice” strategy and on the nature of the social fate attribution. According to Lepianka et al. (2009, pp. 430–431), the social fate category is loosely defined and offers a kind of “all-inclusive” response option for respondents who actually lean toward the choice of other categories. The same authors also rightly point to the lack of a clear theoretical basis for the whole typology and its essentially data-driven nature.

However, there are also arguments in favor of the four-type classification. First, the distinction between “social blame” (SB), “social fate” (SF) and “individual fate” (IF) types allows us to address old concerns about the intermingling of the structural and fatalistic types and their internal division into separate dimensions in some studies (e.g., Furnham, 1982a, 1982b). We believe that the inclusion of the SF category is useful for better delineating the categories of structural and fatalistic attributions as they were originally conceived. SF captures views of poverty that are *not* inherent in these two categories. In essence, agreement with the statement that poverty is “an inevitable part of modern progress” may reflect a belief that the poor are “lagging behind” and constitute a social stratum this is unable to keep up with the development of the modern economy. This aligns broadly with the “culture of poverty” argument, which posits that the poor “are there forever” because poverty is self-perpetuating—through a lack of education, disorganization in everyday life, and a correlated sense of resignation and fatalism (Lewis, 1966, pp. xlii–lii; Harvey and Reed, 1996; Erhard, 2022). To some extent, this interpretation of the “SF” type overlaps with cultural attributions (Cozzarelli et al., 2001) or interactionist attributions (Homan et al., 2017)—both of which appeared as separate dimensions.

A second argument in favor of the four type classification draws from studies that followed the factor analysis approach and included “SF” items, such as “it is an inevitable part of the way the modern world is going,” or “they lack the skills needed in modern working life” (e.g., Niemelä, 2011; da Costa and Dias, 2014; Husz et al., 2022). Interestingly, these explanations enjoy *substantial acceptance among the public*. However, from a methodological perspective, they do not seem to contribute significantly to the structuring of poverty attributions when only two or three factors are extracted. Thus, while research in the factor-analytic tradition has failed to prove the relevance of a “SF” attribution, it has also failed to provide evidence against its relevance. Finally, as a third argument in favor of the four-type classification, some studies (Wright, 1995; Bennett et al., 2016) have concluded that the social fate type is a necessary (and, indeed, logical) adjunct to the usual range of poverty attributions. Although these studies stem from quite different epistemologies and use different concepts and labels, the resulting 2 × 2 matrix of poverty explanations they propose bears a strong resemblance to the four-type classification.

In summary, the four-type classification has been widely used in research, partly because of the brevity of the questions and its inclusion in several international surveys.^1^ Taking account of the criticisms of the forced-choice format, it seems preferable to ask about the four types in separate questions, allowing respondents to rate their agreement with each of the four explanations. This is the case for the SHP, the database for our empirical analyses, which included these questions in three waves (2019–2021). In contrast to the forced-choice approach, this new measure enables researchers to capture the *irrelevance* of poverty attributions, because respondents who believe that none of the provided causes of poverty are “correct” should express minimal agreement with all statements. In addition, the new measure can capture *indifference* (when two or more attributions receive similarly low ratings) and *ambivalence* (when two or more attributions receive similarly high ratings).

## The determinants of poverty attributions

Our aim in this section is to discuss the underlying mechanisms linking social stratification and the formation of poverty attributions and to derive testable hypotheses. Social stratification variables such as income, wealth, social class, occupation, etc. define an individuals’ “objective position in the stratification system” (Kluegel and Smith, 1986, p. 5). This position, together with feelings of relative deprivation among disadvantaged groups, shapes an individual’s propensity to support or oppose different types of social policy and different accounts of poverty. We will focus the theoretical discussion on the agency dimension of poverty explanations, namely on “social blame” (SB) and “individual blame” (IB).

While American research has suggested that lower status individuals often endorse *both* structural and individualistic attributions, European (and other non-American) research has pointed out that stratification variables have a symmetrically opposite effect on the endorsement of the SB and IB attributions.^2^ The lower one’s position in the social hierarchy, the stronger the support for SB explanations and the weaker the support for IB explanations (e.g., Kreidl, 2000; Strapcová, 2005; Davids and Gouws, 2013; Kainu and Niemelä, 2014). Indeed, the distinction between SB and IB is, both conceptually and empirically, the most discriminative to account for inter-individual variation in poverty attributions (Halman and van Oorschot, 1999). This is confirmed in the SHP data, where we also find that SB and IB are farthest apart on the most discriminant component (see Figure A1 in Supplementary material). This distinction is also the most relevant from a policy perspective, as SB and IB are tied to radically different political interpretations of the role of the state in fighting poverty. In contrast, the relationship between fatalistic (IF and SF) attributions and stratification variables is rather unclear from a theoretical and empirical point of view. Therefore, we will formulate our hypotheses in terms of the distinction between SB and IB attributions. Nevertheless, we will also explore the relationship between social stratification variables and fatalistic attributions empirically, to determine whether and how position in the social structure is related to conceptions of poverty as a misfortune beyond the control of individuals and social institutions.

### Mechanisms linking social stratification and poverty attributions

As a systematic theoretical framework on how social stratification may shape the formation of poverty attribution is so far missing in the literature, we first present potential theoretical mechanisms for social stratification more generally, before switching to testable hypotheses.

**Assumption 1: Self-interest**. People tend to agree with causal attributions of poverty which align with their self-interest (Nilson, 1981; Kluegel and Smith, 1983, 1986; Hunt, 1996; Kreidl, 2000; Lepianka, 2007; Kallio and Niemelä, 2014). Essentially, “people in different positions (defined by status, race, gender, or other social distinctions) are expected to react differently to social inequalities that affect them” (Kluegel and Smith, 1986, p. 11). While some people can be said to “benefit” from policies that maintain social inequalities, others would benefit from a reduction in inequalities. Similarly, different groups of people find relief in believing that the consequences of social inequalities are either deserved or unjustified. In short, “people support beliefs from which they profit, and oppose those from which they lose” (Lepianka, 2007, p. 14). On the one hand, individuals who are actual or potential beneficiaries of welfare or anti-poverty policies are expected to support causal attributions which justify their dependence on the welfare state. Thus, “the self-interest hypothesis suggests that the greater support for structural explanations by groups that are disproportionately poor stems from their interest in a type of attribution that justifies their larger dependence on social provisions” (Abts et al., 2023, p. 5). On the other hand, “more privileged groups have a financial interest in preventing a redistributive restructuring of the reward system” (Nilson, 1981, p. 535). Put differently, “individuals in high social positions are usually not interested in having inequalities, from which they profit, reduced” (Kreidl, 2000, p. 156) and can be expected to oppose anti-poverty policies funded by their taxes. This should translate into support for IB explanations and rejection of SB explanations.

**Assumption 2: Self-serving bias**. Poverty attributions have sometimes been analyzed in terms of attribution theory (Nisbett and Ross, 1980; Mezulis et al., 2004). In a nutshell, people tend to attribute their failures to situational/external factors and their successes to dispositional/internal factors. This self-serving bias can in turn enhance or reduce preferences for redistribution (Deffains et al., 2016). In addition, due to an “actor-observer bias” (Jones and Nisbett, 1972; Wilson et al., 1997), people have a tendency to attribute internal causes to the behavior of others and external causes to one’s own behavior. These findings from attribution research may carry over to poverty attributions (Carr and MacLachlan, 1998; Campbell et al., 2001; Hunt and Bullock, 2016; Gugushvili, 2016; but see Vázquez et al., 2017). Individuals in lower social positions are expected to view their underdog status as a personal failure and to favor external causes (Lepianka, 2007, p. 14). In contrast, individuals from more privileged backgrounds, as *observers* of the poverty of others, are expected to ascribe internal causality to the circumstances of the poor.

**Assumption 3: Exposure to poverty**. Several studies postulate that “contacts” with poor individuals and populations play a role in the formation of poverty attributions (Lee et al., 1990; Wilson, 1996; Lee et al., 2004). These contacts can take the form of direct interactions, everyday observations, access to information about the poor, or friendship and family relationships with poor people—all of which can result from one’s own experience of poverty. With some exceptions (e.g., panhandling), exposure to poverty elicits the formation of more positive attitudes toward the poor and “encourages the development of positive emotions and empathy” (Lee et al., 2004, p. 50). In turn, empathetic attitudes should be more widespread among people with lower positions in the social stratification, because such positions are associated with a heightened probability of exposure to poor subpopulations (Hopkins, 2009; Merolla et al., 2011). In contrast, well-off individuals are more likely to live (and work) in an environment insulated from the most visible presence of poor people, reducing empathy and understanding of the structural factors that contribute to poverty.

**Assumption 4: Education**. Similar to exposure, education is likely to cultivate empathy for the poor, although the mechanism is symbolic and intellectual, rather than physical and relational (Hunt, 1996; Guimond, 1999, 2000). However, alternative accounts of the effect of education on the endorsement of structural and individualistic poverty attributions have been proposed in the literature. These are discussed below.

**Assumption 5: Injustice and resentment**. From a different perspective, the main driver of poverty attributions may be seen not as a cold, utilitarian justification for one’s position in society, but rather as “sentiments of loss and injustice due to experienced disadvantages, often directed toward the elite and the other” (Abts et al., 2023, p. 5). Resentment, grievances and perceptions of injustice are expected to fuel SB explanations and to decrease IB explanations (Abts et al., 2023)—although a reverse causality has also been proposed (Schneider and Castillo, 2015). Arguably, people who perceive that they are (or have been) treated unfairly should be drawn mainly from the lower strata. This prediction is broadly consistent with the “justice capital” framework (Thomas, 2022), which defines justice capital as the degree of “access to justice” and is related to social status: “Those in higher economic status have the ability to hire lawyers, purchase insurance, access quality medical care, and have a financial safety net that makes them less vulnerable to unjust circumstances or policy changes” (2022: 191; see also Bartholomaeus, 2025).

**Assumption 6: Ideology.** Unsurprisingly, the vast majority of studies exploring the foundations of poverty attributions have considered the role of ideology, primarily in terms of individuals’ left–right or liberal-conservative orientations (e.g., Miller and Levitin, 1976, pp. 182–184; Furnham, 1982a; Pandey et al., 1982; Williams, 1984; Zucker and Weiner, 1993; Hunt, 2004; Shirazi and Biel, 2005; Larsen, 2006; Brimeyer, 2008; Robinson, 2009; Lepianka et al., 2010; Bobbio et al., 2010; Niemelä, 2011; Weiner et al., 2011; Hunt and Bullock, 2016; Özpinar and Akdede, 2022). With very few exceptions, these studies show that structural attributions are favored by leftists/liberals whereas individual attributions are favored by rightists/conservatives. Indeed, there is hardly any country where social inequalities and poverty have not been politicized and incorporated into the political discourse of the main parties. “Class politics” and “identity politics” are all about the aggregation and mobilization of the interests and claims of particular social strata by specific parties. As a result, different social strata often have distinct ideological positions (Kitschelt, 1993; Bartle, 1998; Kriesi et al., 2008). However, the direction of causality linking ideology and poverty attributions is unclear. Beliefs about poverty are acquired early in life, in the very first stage of political socialization (e.g., Furnham, 1982c; Bullock, 2008; Chafel, 1997; Chafel and Neitzel, 2005; Leahy, 1990), and they could as well be a cause (rather than outcome) of the development of ideology.

Given our focus on social stratification, which in most its aspects is causally antecedent to political attitudes, we will not treat ideology in our main analysis. We thus avoid “overcontrol bias,” which arises when including a variable that lies on the causal path between the two variables of interest (Elwert and Winship, 2014). However, models controlling for left–right self-placements are included in Supplementary material to disentangle the potential effects of ideology from those of self-interest and other mechanisms, and we will address these models in the interpretation of our findings.

### Current financial resources and deprivation

Financial resources are a key aspect of social stratification, and the lack of financial resources (for basic needs) is the key definition of poverty. Self-interest (A1) and self-serving bias (A2) are the main relevant assumptions for the relationship between financial resources and poverty attributions. Highly positioned individuals contribute more to the welfare state than they benefit directly from it and may see their financial success as a result of personal qualities such as hard work, perseverance or intelligence, and see poverty as a lack of these qualities in others. People with few financial resources may be less likely to link the social position to work and merit, but may blame society for their situation (Mack and Lansley, 1985, pp. 205–209; Bullock, 1999; Halman and van Oorschot, 1999; Strapcová, 2005; Kraus et al., 2009; Weiss-Gal et al., 2009; Habibov, 2011; Kainu and Niemelä, 2014). Also, exposure to and empathy toward the poor (A3) and (lack of) personal experiences of injustice (A5) are likely to play a role.

Key indicators for financial resources are income and wealth, as a main source of financing basic needs and defining living standards. However, due to data limitations and methodological reasons, we will focus on income in the empirical analysis.^3^ However, we did test the relationship between wealth (quintiles) and poverty attributions empirically and will comment on them briefly (see Supplementary material for more details). Hence our first hypothesis:

*H1*: The higher a person’s disposable income, the higher her agreement with the IB explanation and the lower her agreement with the SB explanation.

The assumptions of self-interest (A1), self-serving bias (A2) and feelings of injustice (A5) depend on the consciousness of one’s own position. It is therefore not surprising that subjective financial resources have been shown to be more important for poverty attributions than objective position (e.g., for income mobility, Shariff et al., 2016; Mijs et al., 2022). For example, people who do not perceive themselves as poor are unlikely to think of themselves or others like them as part of the poor group when expressing poverty attribution. By the same token, perception of deprivation of goods, activities, and services—e.g., not having a car or not being able to eat out at least once a month for financial reasons (Townsend et al., 1988; Spicker, 2007) may be more relevant to poverty attributions than the purely monetary indicator of income poverty. Moreover, income poverty may amplify the effect of deprivation. The combination of income poverty and deprivation (both as binary variables with a threshold) provides four positions: (1) “Prosperity” is defined by non-poverty (being neither income-poor nor deprived); (2) “precariousness by deprivation” refers to households above the deprivation threshold that are not income poor; (3) “precarious by income” depicts households that are income poor and not deprived; and finally (4) households in (consistent) “poverty” are characterized by both income and deprivation-based poverty.

*H2a*: Financially deprived people are less inclined to provide an IB explanation and more inclined to provide an SB explanation than people without (financial) deprivations.

*H2b*: Persons cumulating financial poverty and financial deprivations (consistent poverty) are less inclined to provide an IB explanation and more inclined to provide an SB explanation compared than individuals who are deprived but not income poor (precariousness by deprivation).

### Past financial resources and income mobility

Not only current financial resources, but also previous experiences during the life course are likely to influence poverty attributions. If a person has experienced poverty in the past, they may be more likely to consider people like themselves when asked to explain poverty (A3). Experiencing a lack of financial resources may rise awareness of structural barriers such as discrimination, lack of access to services or financial experiences (A5), and therefore attribute poverty to social injustice.

*H3*: Persons who experienced financial difficulties in childhood are more inclined to provide a SB explanation and less inclined to provide an IB explanation compared to people who have not experienced financial difficulties in childhood.

Income mobility may have an even stronger effect than current or past financial resources, as individuals tend to justify their changes in position (A2). Gugushvili (2016) argues that upward mobility (especially in the subjective sense of having “done better in life than [one’s] parents”) increases the propensity to make IB attributions, compared to SB attributions. Likewise, Stephenson’s (2000) analysis of Russian and Estonian data shows that an increase in family income fosters individualistic attributions and inhibit structural attributions.

*H4*: Individuals with upward income mobility are inclined to provide an IB explanation and less inclined to provide an SB explanation compared to individuals with stable financial situation or decreasing income mobility.

The hypotheses presented so far are cross-sectional in the sense that they formulate expectations about the *levels* of agreement of different groups of people. From the same theoretical mechanisms, we can also derive dynamic hypotheses about the *change* in agreement within individuals over time, which can be tested with panel data. The dynamic hypotheses for income and deprivation can be formulated as follows:

*H5a*: Individuals with rising (decreasing) income over time increase (decrease) their agreement with the IB explanation and decrease (increase) agreement with the SB explanation.

*H5b*: In years when a person is financially deprived, agreement with SB explanations is higher and agreement with IB explanations is lower than in years when the same person is not financially deprived.

### Education

Education is not only a central element of social stratification, it is also strongly linked to socialization. Accordingly, contradictory expectations have emerged in the literature (see McClosky and Zaller, 1984, pp. 97–100; Kluegel and Smith, 1986, pp. 26, 297–299; Hunt, 1996). On the one hand, education is a factor of economic and social status and, as such, should support individualistic explanations of poverty (IB). For example, better educated individuals may believe that the poor deserve their fate because they have not invested enough time and resources in education. On the other hand, education can have an “enlightening effect,” by fostering attention to low-key information that enables individuals to discern their “real interest,” or by increasing “awareness of inequality and compassion for the disadvantaged” (Hunt, 1996, p. 296). In particular, studies by Guimond and associates offer strong evidence in favor of the socializing effect of education, showing that sustained exposure to particular educational environments modifies the relative importance of individualistic and structural blame attributions, depending on whether these environments endorse the “dominant ideology” of individualism or emphasize the importance of social conditions (Guimond et al., 1989; Guimond and Palmer, 1990; Guimond, 1999, 2000; De Oliveira et al., 2012; Guimond and de la Sablonnière, 2015; see also Smith and Stone, 1989; Sun, 2001; McWha and Carr, 2009).

Likewise, studies suggest that education may reduce the likelihood of making IB attributions of the “laziness” type (Heaven, 1989; Hunt, 1996, 2004; Larsen, 2006; Bray and Schommer-Aikins, 2016). In contrast, the evidence for structural explanations is inconclusive. Overall, the relationship between education and SB explanations has been found to be nonexistent or negative in the United States (e.g., Feagin, 1972; Hunt, 1996, 2004; Robinson, 2009) but possibly positive in Europe (Blomberg et al., 2013; Marquis, 2020). In addition, this relationship may vary over time as a result of changes in the socio-economic context. Clearly, more research is needed to disambiguate results about the role of education and to distinguish its potential stratification effect from its “enlightenment” effect.

*H6*: Individuals with higher educational levels are more inclined to provide a SB explanation and less inclined to provide an IB explanation compared to individuals with lower educational levels.

### Occupation—social class

Occupation is expected to shape poverty attributions through self-interest (A1), self-serving biases (A2), exposure to poverty (A3), or injustice feelings (A5). Most people spend a lot of time in paid work and are exposed to different work environments, cultures and social networks. Jobs have been classified in many different ways. For poverty attributions, the “work logic” is likely to play an important role. Oesch’s (2006) class scheme categorizes occupations into four work logics: interpersonal, organizational, technical and independent. While there are a multitude of possible links with poverty attributions, the theoretical expectations are clearest for the interpersonal and independent work logic. Employees in occupations with an interpersonal work logic may have more contact with vulnerable people, giving them a better understanding of (and empathy for) the situation of the poor (A3, A5). Some occupations also share with poor people an interest in justifying the redistribution system on which their work depends (A1). In contrast, a number of studies have noted a tendency among self-employed individuals to favor IB explanations and/or downplay SB explanations (Larsen, 2006; Paugam et al., 2017, pp. 249–251; Marquis, 2020). This can be equated with self-interest (A1) to the extent that (at least in Switzerland) self-employed workers contribute to the social redistribution system without enjoying social protection in some domains (e.g., unemployment benefits).

*H7a*: Persons in professions with an interpersonal work logic are more inclined to provide a SB explanation and less inclined to provide an IB explanation compared to people with another work logic or inactive individuals.

*H7b*: Self-employed people are more inclined to provide an IB explanation and less inclined to provide an SB explanation compared to people with another work logic or inactive individuals.

Unlike financial resources or education, which are largely exogenous to ideology, the choice of occupations may be more directly influenced by values. Therefore, ideology may be an additional mechanism that explains the potential relationship between occupation and poverty attributions. If, in addition to socialization and ideology, the work logic plays a role, a change in the work logic should be reflected in changing poverty attributions. If we find such dynamic (within) effects, they will provide strong evidence that poverty attributions are driven by occupation or work logic (through A1, A3 or A5), and not by stable characteristics correlated with the work logic.

*H8a*: In years when a person has an occupation with interpersonal work logic, agreement with IB explanations is lower and agreement with SB explanations is higher than in years when the same person does not have an interpersonal work logic.

*H8b*: In years when a person has an occupation with independent work logic, agreement with IB explanations is higher and agreement with SB explanations is lower than in years when the same person does not have an interpersonal work logic.

Occupations are also the basis for the more conventional understanding of vertical social class. As with income, the self-perception may be more important for poverty attributions than external classifications into social class schemes. The class people feel they belong to is likely to shape their poverty attributions. Whether through self-interest (A1), self-serving biases (A2) or injustice feelings (A5), people who perceive themselves as working class or lower middle class are more likely to agree with the SB explanation than middle or upper class people. Upper class people are more likely to agree with the IB explanation and less likely to agree with the SB explanation.

*H9*: The higher one’s perceived social class, the more one agrees with the IB explanation and the less one agrees with SB explanation.

### Place and time: the Swiss context and COVID-19 pandemic

Given the timing of our survey from 2019 to 2021, which covers the COVID-19 pandemic, we will also address the role of period effects. Previous studies have addressed how levels of unemployment, economic growth and poverty rates affect poverty attributions (e.g., Kluegel, 1987; Saunders, 2003; Paugam and Selz, 2005) and, in particular, the impact of economic crises (Hunt and Bullock, 2016; Davidai, 2018; Marquis, 2020). Although the pandemic shared some features of an economic crisis (economic recession, impoverishment, government intervention in the economy to help businesses, etc.), the tragic *health* consequences set the pandemic apart from previous crises that have occurred since the Second World War.

We test our hypotheses using the case of Switzerland, which is a rich country both in terms of both income and wealth. Moreover, the welfare state guarantees a minimum standard of living to its registered residents. The poverty thresholds are relatively high by international standards, because they are based on a common standard of living and make poverty largely invisible. In 2021, official statistics considered residents to be income poor if their equivalized disposable income was less than CHF 2289 (EUR 2474) per month, which affected 8.7 per cent of the population. Using other measures of poverty, in 2021 14.6 per cent of the population lived in relative poverty (income below 60 per cent of the median income) and 5.2 per cent experienced material and social deprivation (Swiss Federal Statistical Office, 2024). Social groups particularly affected by poverty include people living in single and single-parent households, households with no one in work, people with low levels of education and foreigners. Although people aged 65 and over are more likely to be income poor, they do not have a higher poverty rate when assets are taken into account.

## Data, measurements, and methods

### Data

We use data from the Swiss Household Panel (SHP; see Tillmann et al., 2016 for details), an annual survey of a probability-based sample of the population living in private households in Switzerland. All household members aged 14 and over are invited to participate. Although the survey started in 1999, we use the data from the years 2019–2021, which include the attribution of poverty. This three-wave panel contains 37,038 observations from 17,402 individuals. Due to a new refreshment sample (SHP IV) added in 2020, the number of interviews is considerably lower in 2019. 6,713 individuals participated in all three waves, 6,210 in two waves, 4,479 in only one wave. Data were collected each year between September and February of the following year, mainly administered by telephone (80%), the rest being online interviews.

The SHP is well suited to contribute to the literature on poverty attribution. First, the SHP is an interdisciplinary survey that contains detailed information on various aspects of social stratification, including relatively detailed information on income and occupation. Second, the data offer an alternative approach to the forced-choice measure the four-type poverty attributions. Third, the longitudinal data structure allows us to address research questions on individual change. Finally, fixed-effects models which explore within-individual variation are a widely used approach to test or approximate causal influences. If the relationships postulated in the hypotheses are true causal relationships, they should hold not only for differences between individuals, but also for differences in the same individual over time. However, fixed effects models are only suited for (dependent and independent) variables with sufficient within-individual variance. We postulated such relationships for income (hypotheses 5a), deprivation (hypothesis 5b), and occupations (hypotheses 8a, 8b). This approach controls any confounding effects of time-invariant unobserved individual variables that may be related to the observed independent variables. For stable characteristics (education, financial problems in childhood, self-perceived class) we provide only cross-sectional models. Furthermore, we do not estimate fixed effects models for the interaction between deprivation and financial poverty and for subjective income mobility, as results cannot be interpreted straightforwardly.

As with survey data in general, there are different types of non-response in the SHP. Not all households and individuals in the sample participate in the first wave (initial non-response), other individuals drop out in later waves (attrition) and some participants do not answer certain questions (item non-response). Participation in surveys may be linked to poverty attribution. In terms of attrition (dropouts), we see that respondents with higher levels of IB, lower levels of SB or IF are slightly more likely to drop out of the study.

### Dependent variables

While most measures of poverty attributions using the four-types classification follow a *forced choice* between four types of attribution in a single question (see Section 2), the SHP asked about agreement with each of the four types in separate questions. The question starts with a general introduction: *Why are there people living in poverty in this country? Here are four possible reasons. How much do you agree or disagree with each of these four statements explaining why people are poor?* Respondents are then asked to rate their (dis)agreement with each of the four attributions: *Because they are unlucky (IF), Because of laziness and lack of willpower (IB), Because there is injustice in our society (SB), Because it’s an inevitable part of progress (SF).* Respondents receive the questions one after the other in the same order. It is possible that previously asked items influence answers to subsequent items, but this cannot be assessed with the available data. Any potential bias resulting from conditioning would mostly affect the relative importance of the explanations, but is unlikely to impact the relationship with social stratification. Agreement is measured on 11-point scale (0: completely disagree; 5: neither/nor; 10: completely agree).

As can be seen in Table 1, the SHP sample’s mean agreement with the four attributions ranges from 4.3 to 6. This suggests that a substantial part of respondents were in relative agreement with several attribution types. A closer inspection reveals that a majority (52%) of respondents across the three panel waves were in agreement (i.e., ratings in the 6–10 range) with at least two attributions, and 19% were in strong agreement (i.e., ratings in the 8–10 range) with at least two attributions. Overall, the SB type was most favored by the participants (*M* = 6.0), followed by the IF type (*M* = 5.1), the SF type (*M* = 4.6), and the IB type (*M* = 4.3). Not surprisingly, this is more in line with findings usually obtained in Europe than with the individualistic trend found in America. The share of non-response is very low at around 1% for IB, IF and SB, and slightly higher at 2.6% for SF.

Comparing the share of within variance for poverty attributions (between 53 and 63%) with the share of within variance of the explanatory variables (see Table 1), we see that change at the individual level is rather high for poverty attributions. There are several possible reasons for the high within variability. A first is that the measurement of poverty attribution may be unreliable and “noisy” due to measurement error, random variability, ambiguity in respondents attitudes or influence of the measurement conditions. A second reason is that individuals update their perceptions of poverty. Homogeneity is relatively strongest for social blame and heterogeneity strongest for the fatalistic explanations. Table 1 also shows the correlations between the different poverty attributions, which paint a similar picture to that of the principal component analysis in Supplementary material. The correlations are relatively weak, but strongest between SB and IF, and negative between IB and SB.

**Table 1:** Descriptive statistics of dependent variables.

Variable	*n*	Range	Mean	SD (total)	% within	Correlation with		
						SB	IF	SF
Individual blame (IB)	36,547	0–10	4.3	2.6	53%	−0.13	0.10	0.15
Social blame (SB)	36,667	0–10	6.0	2.4	55%		0.20	0.14
Individual fate (IF)	36,580	0–10	5.1	2.7	61%			0.11
Social fate (SF)	36,067	0–10	4.6	2.7	63%			

SD (total) refers to total standard deviation in the pooled data set (between and within variance), % within refers to the share of variance within individuals. NA = Not available. Source: Swiss Household Panel 2019–2021.

### Independent variables and statistical methods

The hypotheses are tested using pooled ordinary least squares (OLS) regression and fixed effects models. In the former, clustered standard errors correct for multiple observations of the same individuals. We apply a complete case analysis, dropping observations with missing values in any of the variables, except for household income, where item non-response is most pronounced, where we use imputed values provided by the SHP. Descriptive statistics are of the variables measuring social stratification and control variables are summarized in Table 2. The question wordings are provided in Supplementary material.

**Table 2:** Descriptive statistics of independent variables.

Variable	*n*	Range	Mean/frequency	SD (total)	% within
Education	37,038		1: Low (14%) 2: Upper secondary (44%) 3: Applied tertiary (16%) 4: Academic tertiary (25%)		3%
Disposable household income (quintiles)	36,996	1–5	3.1	1.4	22%
Financial deprivation	37,038	0, 1	0: No (88%); 1: Yes (12%)	0.3	38%
Change of the financial situation (from age 18)	28,673	0–10	5.3	1.4	76%
Financial problems in childhood	37,038	0–2	0.26	0.6	0%
Perceived social class (only measured in 2020 from age 16)	14,806		0: None/other (66%) 1: Working class (3%) 2: Lower middle class (5%) 3: Middle class (15.3%) 4: Upper middle class (9.6%)	1.5	0%
Oesch class scheme	36,282		0: No past job (35.3%) 1: Large employers (0.9%) 2: Self-employed professionals (2.3%) 3: Small business owners with employees (2.3%) 4: Small business owners with employees (4.1%) 5: Technical experts (3.9%) 6: Technicians (3.4%) 7: Skilled manual (5.7%) 8: Low-skilled manual (1.2%) 9: Higher-grade managers and administrators (7.6%) 10: Lower-grade managers and administrators (3.4%) 11: Skilled clerks (8.0%) 12: Unskilled clerks (0.5%) 13: Socio-cultural professionals (4.2%) 14: Socio-cultural semi-professionals (8.0%) 15: Skilled service (5.4%) 16: Low-skilled service (3.9%)	0.5	21%
Gender	37,038	0,1	0: Women (53%) 1: Men (47%)	0.5	Very small
Age group	37,038		1: Less than 25 yrs. (12%) 2: 25–34 yrs. (12%) 3: 35–44 yrs. (11%) 4: 45–54 yrs. (17%) 5: 55–64 yrs. (19%) 6: 65–74 yrs. (16%) 7: 75 yrs. and more (12%)		1.5%
Nationality	37,038	0,1	0: Foreigner (6%) 1: Swiss (94%)	0.28	Very small
Year	37,038	2019–2021	2019: 8,789 (36%) 2020: 15,439 (33%) 2021: 12,810 (31%)		
Survey mode	37,038	0,1	0: Telephone (92%) 1: Web (8%)	0.3	16%

SD (total) refers to total standard deviation in the pooled data set (between and within variance), % within refers to the share of variance within individuals. NA = Not available. Source: Swiss Household Panel 2019–2021.

We estimate four different models, each offering a perspective on social stratification from a different angle. The first model (financial resources model) includes educational level, household income and deprivation to capture the current financial situation. *Educational level* is coded into four levels (low, upper secondary, tertiary vocational track, tertiary academic track).^4^ *Income* is included as quintiles of household disposable income (equalized using the modified OECD scale; top-coded at 99%; missing values imputed). For *deprivation*, we use a dichotomous variable indicating whether the household experiences at least two deprivations from a list of 12 items (see Supplementary material for details). We also include an interaction between the lowest income quintile and deprivation, to distinguish between consistent poverty, precariousness by income, precariousness by deprivation and prosperity. An alternative model including *wealth* is presented in Supplementary material.

The second model (income mobility model) looks at how the past income situation is evaluated. We include a measure of subjective income mobility since the previous year (assessment of whether the financial situation has improved or worsened on a scale from 0 to 10, where 5 is no change). We also test whether respondents indicated financial problems in youth (1 yes, 0 no). Education is also included in this model in order to separate financial and educational aspects.

The third model (social class model) distinguishes social classes based on ISCO categorization and self-perceived social class. The Oesch class scheme (Oesch, 2006) distinguishes 16 categories. The four (horizontal) work logics (technical, organizational, interpersonal and independent) are divided vertically into four levels of marketable skills: Professional/managerial; associate professional/managerial; generally/vocationally skilled; low/un-skilled. For the inactive, information on their last occupation was used. To measure perceived social class, respondents were asked (only in 2020) whether they felt they belonged to a particular social class. Those who answered “no” (63%) and those who do not know which class to name (3%) were attributed to the “none” category.^5^ Educational levels are not included in this model, as they are closely related to the vertical dimension of the class scheme.

The fourth model (fixed effects model) analyses how changes in income, deprivation and occupations are related to changes in poverty attributions. Variables which are highly stable (education) or have been measured only once (perceived social class) are not part of the fixed effects model.

All models include the year of observation (2019, 2020, 2021) to capture period effects and a limited number of control variables: age (recoded into seven age categories: 14–24, 25–34, 35–44, 45–54, 55–64, 65–74, and 75 and older), gender and nationality (Swiss, others), and interview mode, because modes administered by interviewers yield more socially desirable results than self-administered surveys (Klausch et al., 2013). For the fixed effects model, only time-varying controls (year dummies, nationality, survey mode) are included as controls. Each of the four models is estimated separately for the four poverty explanations, leading to a total of 16 estimated models. To facilitate comparisons across categories of variables, Supplementary material includes an alternative presentation of the regression results with predicted probabilities. As discussed, we do not include political attitudes or ideology as controls in the main models, as they might be moderators, mediators or colliders and have a high potential of reverse causality in the main models (see Vaisey and Miles, 2014, for statistical aspects; Kluegel and Smith, 1986, for the causal flow running from individuals’ background characteristics to their policy attitudes). The alternative specification for all models including left–right placement are shown in Supplementary material.

## Results

### Poverty attributions over time and the COVID-19 pandemic

**Figure 2:**
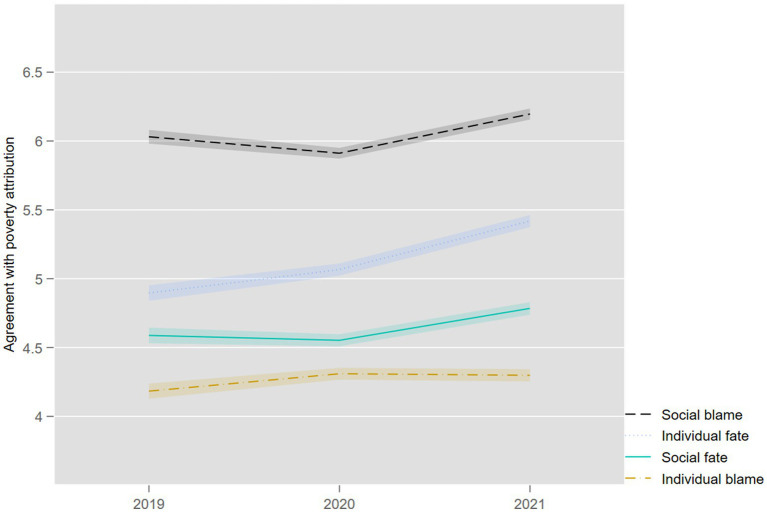
Evolution of poverty attributions, 2019–2021 (truncated axis, mean values). Source: Swiss Household Panel (SHP).

Before testing the hypotheses on social stratification, we briefly discuss the prevalence of poverty attributions and changes over time (within individuals and period effects). Figure 2 shows poverty attributions from 2019 to 2021, controlling for survey mode. The pattern of support for the four attribution types has remained stable over the years, with SB being the most prominent explanation, followed by IF, SF, and IB explanations. IB scores relatively low in Switzerland, suggesting that blaming the poor for their poverty is not as widespread as in other countries. At a finer scale, however, the evolution of poverty attributions over time shows interesting changes in the context of the COVID-19 pandemic. While most poverty attributions have remained relatively stable over time (less than 0.2 points increase), the individual fatalistic explanation (“bad luck,” IF) has progressed considerably between 2019 and 2021 (by almost 0.6 points).

It is perhaps worth repeating that the SHP fieldwork in 2019 took place just before the outbreak of the COVID-19 pandemic (September 2019–February 2020), while the two following panel waves in 2020 and 2021 took place at the height of the pandemic. It is therefore probably no coincidence that the COVID-19 pandemic affected poverty attributions mainly through its IF dimension. Conceptually, poverty has often been viewed as a pathology (Goldsmith and Blakely, 2010; Fitzpatrick, 2011). However, unlike other causes of health problems that are reminiscent of the IB or SB categories (e.g., addictions such as smoking or alcohol consumption, exposure to air pollution or contaminated food products), viral infection is a typically fatalistic cause. Although few questions referred to the COVID-19 pandemic, considerations related to the pandemic were arguably very salient to respondents and likely primed their evaluations of poverty causes. Some studies are broadly supportive of this claim (e.g., Wiwad et al., 2021; Nandori, 2021; Van Hootegem and Laenen, 2023).

### Financial resources model and education

Table 3 shows the results of the Ordinary Least Square (OLS) regression models with pooled data (from 2019 to 2021), showing how individuals differ in terms of poverty attributions with respect to their level of education and current financial resources. The explanatory power of the OLS models in terms of explained variance is moderate. The R-squared is highest for IB (4.0%), and lower for SB (2.0%), IF (3.2%) and SF (2.8%). Socio-economic and demographic variables therefore explain only a small part of the variation in poverty attributions.

**Table 3:** Financial resources model (OLS coefficients).

	IB		SB		IF		SF	
Educational level (Ref: low)								
Upper secondary	−0.106		0.095		0.099		−0.112	*
Applied tertiary	−0.279	**	0.050		0.156	*	−0.306	**
Academic tertiary	−1.029	**	0.420	**	0.658	**	−0.784	**
Income quintile: (Ref: 3rd)								
1. Quintile	0.117	*	−0.023		−0.066		0.134	*
2. Quintile	−0.016		0.023		−0.009		−0.021	
4. Quintile	0.030		−0.028		0.131	**	−0.055	
5. Quintile	0.076		−0.295	**	0.126	*	−0.136	**
Deprivation	−0.136	*	0.471	**	−0.046		0.103	
First income quintile*deprivation	−0.394	**	0.037		0.152		−0.185	
Age (Ref: younger than 25)								
25–34	0.587	**	0.031		0.375	**	0.087	
35–44	0.512	**	−0.024		0.574	**	0.148	*
45–54	0.169	*	−0.172	**	0.477	**	0.080	
55–64	0.037		0.025		0.583	**	0.222	**
65–74	0.130		0.094		0.850	**	0.477	**
75 and more	0.349	**	0.032		1.043	**	0.915	**
Swiss nationality	−0.360	**	0.300	**	0.331	**	−0.058	
Men (Ref: women)	0.714	**	−0.354	**	0.026		0.103	**
Survey mode: Web (Ref: telephone)	−0.068		0.137	**	−0.302	**	0.017	
year/wave of data collection								
2020	0.141	**	−0.113	**	0.166	**	−0.032	
2021	0.148	**	0.166	**	0.492	**	0.190	**
Number of observations	36,510		36,631		36,543		36,025	
Adjusted R-squared	0.04		0.02		0.03		0.03	

Dependent variables: agreement with poverty attributions. ** : *p* < 0.01; *: *p* < 0.05. Source: Swiss Household Panel (SHP).

**Figure 3:**
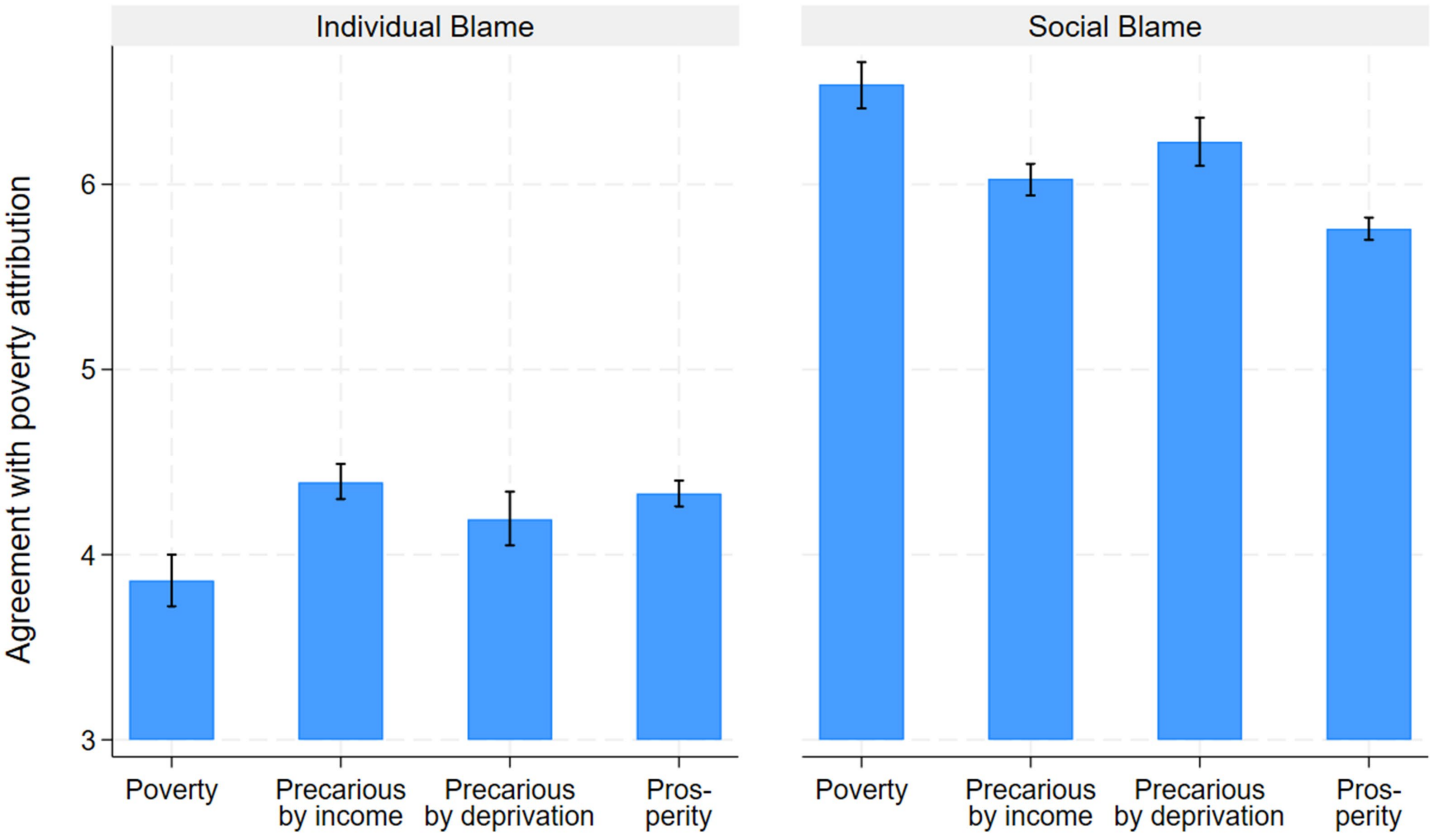
Predicted poverty attribution by income and deprivation (interaction). Truncated axis, 95% confidence intervals. Source: SHP 2019–2021.

For financial resources, the interaction term must be taken into account when interpreting the coefficients. Figure 3 shows the predicted poverty attribution for the four positions in terms of precariousness by deprivation and income. As concerns the IB explanation, the coefficients for income quintile and the similar prediction for individuals in prosperity and in financial precariousness show that household income is not significantly related to the laziness argument. For SB, there is an effect for respondents in the highest income quintile, which are least likely to agree with the social justice argument. Surprisingly, low income is not related to agreement with SB. Support for hypothesis 1 is therefore very weak. However, the results for wealth point in the expected direction for financial resources (see Supplementary material). For IB, the highest quintile agrees most strongly with the laziness explanation, while low wealth is unrelated to it. For SB, the relationship is more or less linear, with the lowest quintile showing the strongest agreement with the social injustice argument and the highest quintile showing agreeing the least.

Deprivation is related to both IB and the SB explanations. As postulated in Hypothesis 2a, deprived people are less likely to explain poverty by laziness and more likely to explain poverty by social injustice. Moreover, consistent poverty (compared to precariousness due to either deprivation or income) further reduces agreement with the claim that the poor are lazy (see Figure 3). This shows that the cumulation of income and deprivation is crucial for predicting IB. For the injustice argument, however, there is no additional interaction effect from this combination. We therefore find partial support for H2b.

Interestingly, financial resources are also associated with fatalistic poverty attributions. Although we did not formulate an explicit hypothesis, it is somewhat surprising that people in favorable positions (higher household income and no deprivation) show the strongest agreement with the “bad luck” (IF) explanation. The SF explanation is mostly unrelated to the financial situation.

Regarding education, we find a clear socialization effect in line with H6. Interestingly, this effect is mainly due to the distinction between the academic and the vocational educational tracks, with the latter passing through apprenticeships. Only individuals with an academic tertiary education are more likely to perceive poor people as victims of social injustice (SB) than individuals with a lower educational level. Those with a tertiary education in the occupational track show similar agreement with SB. For IB, vertical educational level plays a role, with higher educated people being less likely to consider poor people lazy (IB). However, again, this effect is much smaller for applied tertiary education than for academic tertiary education. The low acceptance of the “laziness” explanation among the tertiary educated is in fact one of the strongest predictors of poverty explanations overall, with a difference of about 1 point on the scale. These results can be interpreted as further indications in favor of the socialization or enlightening effect of education rather than a social stratification effect.

Interestingly, education is also strongly associated with fatalistic explanations. Those with tertiary education are more likely to attribute poverty to “bad luck” than those with compulsory or upper secondary education, but again, agreement is strongest for those with academic tertiary education. Indeed, this finding runs counter to previous studies, which tend to show that IF explanations are more popular among less educated respondents (Feather, 1974; Halman and van Oorschot, 1999; Hunt, 2004; Blomberg et al., 2013; Gugushvili, 2016). We find this pattern for SF explanations though: people with lower levels of education are more likely to agree that “poverty is inevitable in modern societies.”

When controlling for left–right position, the conclusions from the financial resources model remain largely unchanged, although coefficients of tertiary education become considerably smaller, and income is no longer significantly associated with IB. This could indicate that political ideology might mediate part of the effect of academic tertiary education, or that self-selection into academic tertiary education plays a role. In contrast, controlling for left–right position hardly affects the coefficients of fatalistic explanations.

### Income-mobility model

In the income mobility model (see Table 4), we test whether (self-reported) financial problems in adolescence and income change are associated with poverty attributions. While respondents who experienced financial problems in their childhood or adolescence are more likely to agree with the SB explanation of poverty, although the effect is weak (0.1 difference on the scale), there is no difference for the IB explanation. Respondents who report that their financial situation has improved (worsened) in the last year are more (less) likely to agree with the laziness explanation and less (more) likely to agree with the social injustice explanation. To illustrate the effect size, the predicted score for laziness is 4.0 for strongly deteriorated, 4.2 for no change and 4.5 for strongly improved. In summary, H3 is supported for SB (but not for IB) and H4 is supported for both SB and IB. When controlling for left–right positions, all coefficients of the income mobility model remain significant, and the association between financial problem in youth and IB reaches significance.

**Table 4:** Income mobility model (OLS coefficients).

	IB		SB		IF		SF	
Financial problem in youth	0.044		0.123	**	−0.064	*	0.010	
Change in financial situation	0.054	**	−0.073	**	0.000		−0.014	
Educational level (Ref: low)								
Upper secondary	−0.133	*	0.010		0.134	*	−0.161	**
Applied tertiary	−0.301	**	−0.067		0.201	*	−0.343	**
Academic tertiary	−1.071	**	0.281	**	0.681	**	−0.881	**
Age								
25–34	0.501	**	0.061		0.389	**	0.120	
35–44	0.512	**	−0.001		0.551	**	0.136	
45–54	0.160	*	−0.194	**	0.468	**	0.105	
55–64	0.036		−0.015		0.603	**	0.275	**
65–74	0.133		0.052		0.812	**	0.524	**
75 and more	0.309	**	−0.022		0.970	**	0.977	**
Swiss nationality	−0.219	**	0.258	**	0.405	**	0.022	
Men (Ref: women)	0.731	**	−0.324	**	0.018		0.096	*
Survey mode: cawi (Ref: CATI)	−0.128	*	0.159	**	−0.383	**	−0.021	
year/wave of data collection								
20	0.075	*	−0.117	**	0.190	**	−0.062	
21	0.163	**	0.156	**	0.508	**	0.204	**
Number of observations	28,338		28,413		28,386		28,023	
Adjusted R-squared	0.04		0.01		0.03		0.03	

Dependent variables: agreement with poverty attributions. ** : *p* < 0.01; *: *p* < 0.05. Source: Swiss Household Panel (SHP).

### Social class model

Self-perceived social class is weakly associated with poverty attributions (see Table 5). Most importantly, the minority of respondents who think of themselves as belonging to a social class are less likely to agree with the laziness explanation and more likely to agree with the social injustice explanation. One possible reason is that awareness for social stratification correlates with both self-assignment of social class and perception of the poor. Among those who place themselves within a social class, there are only weak differences regarding poverty attributions, with most differences being insignificant. The exception is that the lower middle class is more likely to agree with the SB explanation than the middle and upper middle classes (see Table A3 in Supplementary material for predicted agreement for each category). Although this points to the expected effect, there is little overall support for hypotheses 9.^6^

**Table 5:** Social class model (OLS coefficients).

	IB		SB		IF		SF	
Perceived social class (Ref: None)								
Working class	−0.116		0.431	**	0.263		0.287	*
Lower middle class	−0.279	**	0.624	**	0.158		−0.143	
Middle class	−0.160	*	0.312	**	0.427	**	−0.113	
Upper middle class	−0.306	**	0.200	**	0.737	**	−0.132	
Occupational class position (Ref: inactive)								
*Independent work logic*								
Large employers	0.432	*	−0.620	**	−0.233		−0.456	
Self-employed professionals	−0.386	**	−0.128		0.221		−0.476	**
Small business owners with employees	0.768	**	−0.375	**	−0.135		−0.166	
Small business owners without employees	0.254	*	−0.264	*	−0.158		−0.152	
*Technical work logic*								
Technical experts	−0.052		0.088		0.432	**	−0.681	**
Technicians	0.440	**	−0.175		−0.126		−0.341	*
Skilled manual	0.943	**	−0.267	**	−0.467	**	0.135	
Low-skilled manual	0.759	**	−0.162		−0.420		0.261	
*Organizational work logic*								
Higher-grade managers and administrators	0.183		−0.364	**	0.196		−0.428	**
Lower-grade managers and administrators	0.483	**	−0.336	**	0.015		−0.151	
Skilled clerks	0.530	**	−0.246	**	−0.119		−0.079	
Unskilled clerks	−0.369		0.622		−0.161		−0.349	
*Interpersonal work logic*								
Socio-cultural professionals	−0.810	**	0.528	**	0.428	**	−0.776	**
Socio-cultural semi-professionals	−0.389	**	0.137		0.050		−0.401	**
Skilled service	0.606	**	−0.291	**	−0.283	*	0.107	
Low-skilled service	0.550	**	−0.051		−0.121		0.160	
Age								
25–34	0.385	**	0.098		0.414	**	−0.137	
35–44	0.199	*	0.019		0.590	**	0.034	
45–54	−0.125		−0.005		0.515	**	−0.049	
55–64	−0.158		0.093		0.626	**	0.020	
65–74	0.128		0.075		0.837	**	0.233	*
75 and more	0.428	**	−0.080		1.046	**	0.711	**
Swiss nationality	−0.300	**	0.260	**	0.199	*	−0.110	
Men (Ref: women)	0.490	**	−0.299	**	0.056		0.070	
Survey mode: cawi (Ref: CATI)	0.034		0.126	*	−0.235	**	−0.026	
Number of observations	14,100		14,312		14,253		14,070	
Adjusted R-squared	0.04		0.02		0.03		0.02	

Dependent variables: agreement with poverty attributions. ** : *p* < 0.01; *: *p* < 0.05. Source: Swiss Household Panel (SHP).

Current occupations do show an association with poverty attributions (see Table A3 in Supplementary material for predicted values). The differences between occupations are rather large for IB. Skilled and low skilled manual workers, as well as small business owners with employees show the highest agreement with the laziness explanation (predicted values amount to between 4.9 and 5.1), followed by skilled and low-skilled service workers and skilled clerks (predicted value of 4.7), large employers, technicians and lower-grade managers and administrators (predicted values of 4.6). Support for IB is weakest among socio-cultural specialists (predicted value of 3.4) followed by socio-cultural semi-professionals and unskilled clerks (predicted value of 3.8). For SB, we find that occupations that score high on IB tend to score low on SB and vice versa, but the differences between occupations are somewhat smaller. The highest levels of agreement with SB is found among unskilled clerks (6.6), socio-cultural professionals (6.5) and socio-cultural semi-professionals (6.1). The lowest levels is found among large employers (5.4), small business owners with employees (5.6) as well as lower and higher grade managers and administrations and small business owners without employees (5.7).

Taken together, the results for occupations confirm that the work logic does play a role, but it does not fit exactly into the categorization used in the class scheme. For example, the interpersonal work logic consists of socio-cultural and service workers. While the former score very high on SB and very low on IB, skilled workers are positioned at the opposite. This difference may be due to selection (people who think that the system is unfair may be more likely to choose a job as a socio-cultural specialist) or to the nature of work, which is quite different for socio-cultural professionals and service workers (e.g., the self-interest assumption holds only for socio-cultural professionals). Hypothesis 7a is clearly supported for socio-cultural professionals, but not for service workers, even though both are classified in the interpersonal work logic. Interestingly, unskilled clerks are close to socio-cultural specialists in terms of their poverty attributions.

A similar observation can be made for the independent work logic, where we predicted low agreement for SB and high agreement for IB. Indeed, we find this for small business owners with employees and large employers, and to a lesser extent for small business owners without employees, thus overall supporting hypothesis 7b. However, the self-employed professionals are very different, particularly for IB. This may be due professions such as medical doctors, psychologists or lawyers, where interpersonal work logic plays an important role, although these occupations are classified in the independent work logic. Nevertheless, hypothesis 8b is largely supported.

There are also some associations between the occupations and fatalistic explanations, although the differences are smaller than for the agency dimension. While generally, the IF explanation shows a similar pattern as the SB explanation, some exceptions are worth mentioning. Technical experts show average agreement for IB and SB; in contrast, their tendency to agree with the “bad luck” explanation (IF) and their disagree with the “poverty inevitable” explanation (SF) is pronounced. While unskilled clerks showed a pattern similar to socio-cultural specialists for IB and SB, this does not apply to IF, with which low-skilled clerks tend to disagree.

Interestingly, not only the current occupation but also parents’ occupation are related to poverty attributions, with effects almost as important as for current occupations, at least for some professions. Results for model using parent’s social class are shown in Supplementary material.

### Fixed-effects analysis (within perspective)

The final model to be discussed is the fixed effects model (see Table 6). The main result is that the within-effects are close to zero and insignificant; changes in individuals’ income or deprivation do not result in significant changes in their poverty attribution and cannot explain short-term volatility. Although explained variance (R squared) is not a crucial criterion for model quality, a value of zero explained variance underlines that stratification variables do not contribute to explaining the rather strong variability in poverty attributions over time. Furthermore, shifts in the left–right position cannot explain within-individual variation (see Supplementary material). The only significant coefficients in the model are about the effects of two occupations on IB attributions. Workers who change to (leave) a skilled manual profession are likely to increase (decrease) their agreement with the laziness explanation. The same applies, to a smaller extent to jobs in the skilled service category. As manual workers do not have an interpersonal work logic, this is largely in line with the expectation and finding from the cross-sectional model. For service workers, however, this is contrary to the expectation that interpersonal work-logic is related to low agreement with the laziness explanation, but in line with findings from the cross-sectional model. For SB, a change of occupation has no effect. Interestingly, changing to a job with an interpersonal work logic does not reduce agreement with IB or increase agreement with SB. This suggests that the poverty attributions of socio-cultural professionals observed in the cross-sectional model are more likely to be due to selection rather than personal experience.

**Table 6:** Fixed effects model (regression coefficients).

	IB		SB		IF		SF	
Income quintile: (Ref: 3rd)								
1. Quintile	−0.043		0.013		−0.133		0.084	
2. Quintile	−0.063		0.016		−0.108		−0.124	*
4. Quintile	0.042		0.072		0.072		−0.005	
5. Quintile	−0.045		−0.099		−0.009		−0.126	
Deprivation	0.018		−0.045		0.112		0.060	
Oesch class position—16 classes								
*Independent work logic*								
Large employers	0.355		0.227		0.155		0.232	
Self-employed professionals	−0.203		0.044		0.248		−0.116	
Small business owners with employees	0.200		−0.011		0.250		0.151	
Small business owners without employees	−0.053		0.074		0.181		0.080	
*Technical work logic*								
Technical experts	0.105		0.293		0.280		−0.237	
Technicians	0.098		0.151		0.465	*	−0.216	
Skilled manual	0.250		0.237		0.357	*	0.072	
Low-skilled manual	0.737	**	0.244		−0.047		−0.388	
*Organizational work logic*								
Higher-grade managers and administrators	0.248		0.226		0.758	**	−0.113	
Lower-grade managers and administrators	0.045		0.031		0.106		0.120	
Skilled clerks	0.158		0.102		0.149		0.153	
Unskilled clerks	0.113		−0.443		−0.020		0.395	
*Interpersonal work logic*								
Socio-cultural professionals	0.041		0.232		0.457	*	0.048	
Socio-cultural semi-professionals	0.202		0.045		0.222		−0.043	
Skilled service	0.284	*	0.133		0.127		0.208	
Low-skilled service	0.099		0.031		−0.077		−0.115	
Age								
25–34	−0.182		−0.013		−0.201		0.181	
35–44	−0.189		−0.064		−0.343		0.296	
45–54	0.113		0.092		−0.380		0.438	
55–64	0.323		0.084		−0.454		0.541	
65–74	0.532		0.080		−0.219		0.609	
75 and more	0.596		−0.009		−0.147		0.578	
Swiss nationality	−0.511		0.231		0.109		0.372	
Survey mode: cawi (Ref: CATI)	−0.083		0.334	**	−0.281	**	−0.192	*
Year/wave of data collection								
20	0.039		−0.138	**	0.223	**	−0.071	*
21	0.087	*	0.124	**	0.551	**	0.156	**
Number of observations	35,308		35,433		35,348		34,849	
Adjusted R-squared	0		0		0		0	

Dependent variables: agreement with poverty attributions. ** : *p* < 0.01; *: *p* < 0.05. Source: Swiss Household Panel (SHP).

Despite lacking support for hypotheses 8a and 8b for the within-models, it would be wrong to conclude that poverty attributions are unaffected by the work logic. Some specific work environments (low-skilled manual and skilled service occupations) are linked to IB, although this should not be overinterpreted due to potential type II error of testing many occupations. More importantly, certain changes in occupation are associated with agreement to IF. The occupations that stand out are not specifically related to work logic, but rather to qualified employees (working as a technician, skilled manual, higher grade manager and administrators, socio-cultural professionals).

## Discussion

The aim of this contribution was to explore the relationship between causal attributions of poverty and social stratification. We used the four-type classification of poverty attributions distinguishing “individual blame” (IB), “individual fate” (IF), “social blame” (SB) and “social fate” (SF). This study is the first to systematically test the relationship with social stratification based on theoretical mechanisms and to incorporate an encompassing view of social stratification.

Data were collected separately for each explanation in three panel waves in 2019, 2020, and 2021. Agreement was strongest for the “social injustice” (SB) explanation, followed by “bad luck” (IF), “inevitable part of modern progress” (SF) and finally “laziness and lack of willpower” (SF). Considering that Switzerland is a rich country that attaches great importance to individual responsibility, the low level of agreement with the IB statement is remarkable. At the same time, the strong support of the SB explanation corresponds to the generous welfare state in Switzerland and in Europe more generally, from a comparative perspective. The most striking effect is the rise in fatalistic explanations during the COVID-19 pandemic, which is not related to personal changes in economic circumstances. It will be the task of further research to explore the impact of the pandemic more closely.

We find that poverty attributions are related to social stratification, however the link is weak. Socio-demographic characteristics can only explain a small portion of the variation in poverty attributions between individuals (OLS models) and cannot explain within-individual variance in poverty attributions in the short-term (FE models). Nevertheless, social stratification helps to describe differences between population groups and the results from the empirical analysis contribute to improve our knowledge on the formation of poverty attributions and the mechanisms in place.

The results for income, wealth and deprivation allow conclusions to be drawn about the theoretical mechanisms that determine poverty attributions. The assumption of *self-interest* seems to play an important role in agency-explanations of poverty. Being in the lowest income quintile, and experiencing deprivation is related to agreement with the IB explanation; conversely, being in the highest wealth quintile is related to low agreement with the IB explanation. High income and wealth, and no material deprivation, are related to low agreement with the SB explanation. Similarly, being in the highest income or wealth quintile and no material deprivation is related to low agreement with the SB explanation.

These results also largely align with the *self-serving bias* mechanism. In addition, this mechanism best explains the findings for (perceived) income mobility. Individuals who state that their financial situation has improved, are more likely to agree with the IB explanation, while individuals who state that their financial situation has deteriorated are morel likely to agree with the SB explanation, thus attributing success to their own behavior and failure to society.

The fact that results are clearer for deprivation and perceived change in income than for the purely financial aspect suggest that the mechanisms of self-interest and self-serving bias are not “mechanical” but require a self-perception of one’s position in the social stratification. The results for perceived social class can be interpreted in a similar way: the mere ability or willingness to place oneself in a (vertical) social class is more important for poverty attributions than the placement itself.

*Exposure to poverty* seems to play only a small role in poverty attributions. Having a low income is not related to SB, and only affects IB if it is combined with deprivation. However, the fact that deprivation is associated with both IB and SB, and that respondents who experienced financial problems in their childhood or youth report higher levels of agreement with SB, could be a consequence of exposure to poverty. Nevertheless, financial problems in childhood do not affect IB. This suggests that memories of past hardship trigger awareness of social responsibility for poverty but do not dilute individual responsibility. The findings regarding deprivation and financial problems in youth could also reflect *experience of injustice and resentment*.

Our analysis clearly indicates the presence of strong socialization effects. We found a strong relationship between poverty attributions and education. Higher educational levels are associated with greater agreement with SB and less agreement with IB. Interestingly, this effect mainly be attributed to the difference between academic and vocational educational tracks in Switzerland. Those with an academic tertiary education are the most likely to agree with explanations that view poor people as victims of social injustice (SB), and least likely to blame them (IB). In addition, the fixed effects models showed that short-term volatility in poverty attribution is not influenced by (changing) financial resources or deprivation. Therefore, socialization and dispositions are likely to be primary determinants of poverty attributions, while current life conditions play a minor role. This is in line with studies on other political attitudes or political trust, which demonstrate limited influence of life experiences (Devine and Valgarðsson, 2024). Finally, the strong effects found for parental occupation on poverty attributions suggest that primary socialization should not be overlooked.

We also analyzed the relationship between social class and poverty attribution, focusing on the work logic. We expected the interpersonal work logic to be linked to high agreement with SB and low agreement with IB. This effect was observed for socio-cultural professionals and semi-professionals in the cross-sectional model, but not in the fixed effects model. Furthermore, we found that the independent work logic is linked to poverty attributions. Small business owners and large employers showed relatively high agreement with SB and low agreement with IB. The same applies to unskilled clerks. Again, these results were not confirmed in the fixed effects model. Therefore, self-interest, exposure to poverty and resentment are unlikely to explain the poverty attributions of socio-cultural professionals and those in an independent work logic. Rather, their poverty attributions are likely to reflect socialization and ideology. This interpretation is further backed by the finding that service workers, who also follow an interpersonal work logic, exhibit a different pattern from socio-cultural professionals and by the strong association between parent’s social class and poverty attributions by their children.

It is also important to note that occupations were also the only significant stratification variables in the fixed effects models. While we could not find generalizable patterns that changes in the social stratification affect poverty attributions, the findings for occupation for specific contexts show that poverty attributions are not immune to life experiences. The way how experiences shapes someones view might be too heterogenous and individual to be generalized with statistical models. At the same time, the collective experience of the COVID-19 pandemic had a notable effect on the population.

Our analysis also covered fatalistic poverty attributions. Generally, IF tends to follow a similar pattern as the SB explanation, although the increasing agreement for IF with income presents an important exception. The SF explanations is hardly associated with social stratification. It is a task for future research to present a theoretical basis for the determinants of fatalistic explanations and to evaluate the role of SF in the structure of poverty attributions. To our knowledge, this is the first study that explicitly analyses individual dynamics of poverty attributions. We found that poverty attribution are rather volatile, but were not able to explain short-term changes in poverty attributions by financial resources, occupations or left–right position. It remains a task of future studies to find out whether poverty attribution measures are have a high stochastic component, or whether other variables are better suited to explain why poverty attributions change over time.

## Data Availability

Publicly available datasets were analyzed in this study. This data can be found at: https://www.swissubase.ch/fr/catalogue/studies/6097/20179/overview.

## Footnotes

^1^Here is a sample of studies published in recent years: Blomberg et al. (2013), Kainu and Niemelä (2014), Corcoran et al. (2015), Gugushvili (2016), de Vries (2017), Harell et al. (2017), Wu and Chou (2017), Steckermeier and Delhey (2019), Marquis (2020), Wong (2020), Gugushvili et al. (2021), Marquis and Rosset (2021), van Hootegem et al. (2024).

^2^US studies strongly suggest that people of lower status are more likely to support structural explanations of poverty. However, evidence for individualistic explanations is mixed. Studies have generally found no substantial effect of social status on the preferences for individualistic attributions (e.g., Bullock, 1999, 2004; Robinson, 2009; Hunt, 1996, 2004; Kluegel and Smith, 1986; Kluegel, 1987; but see Kraus et al., 2009 for an exception). American scholars tend to explain this by the “dominant ideology” of individualism which pervades all sectors of American society, including the more disadvantaged groups who are deeply ambivalent about the causes of poverty and wealth (Hochschild, 1981; Kluegel and Smith, 1986; Robinson, 2009). As a result of this ideological “indoctrination” (e.g., Jost and Banaji, 1994; Jost, 2020), a phenomenon of “split consciousness” (Kluegel et al., 1995) or “dual consciousness” (Hunt, 1996, 2016; Bullock and Waugh, 2005; Merolla et al., 2011) has arguably developed among the lower classes.

^3^First, wealth measures are only available for one year and a subsample of households interviewed (collected in 2020, but not for subsample SHP IV). Second, the share of item non-response is quite high and the wealth measures in the SHP are approximate. Third, income, wealth and deprivation are correlated and the inclusion of too many variables risk to overburden the models. We considered testing the interaction between income and deprivation as more interesting from a theoretical perspective, because the underlying mechanisms for income and wealth are highly similar. Fourth, income is of particular interest for our analysis, because income can change over short periods of time and is potentially relevant in the COVID-19 context.

^4^Low education (1): incomplete or completed compulsory school, 1 year school of commerce or domestic science; Upper Secondary (2): general training school, apprenticeship, high school; Vocational tertiary (3): vocational high school; Academic tertiary (4): Teacher education, university of applied sciences, university academic high school, PhD.

^5^A follow-up question asked respondents about which class they belong to if they have to choose, which we did not consider as to reflect a self-perception. With the forced choice, only 5 percent would be left without attribution.

^6^It has to be taken into account that not only the coefficient size, but also limited number of observations are responsible for non-significant effects. Self-perceived class was only collected in 2020, and very few respondents of the sample assigned themselves into the working class (*n* = 454; 3%), or lower middle class (*n* = 773; 5%).
