# Supplementary material for: Causal attributions of poverty: a social stratification analysis
Source: Front Sociol. 2025 Jun 10;10:1591235. doi: 10.3389/fsoc.2025.1591235 (PMC12185453; doi:10.3389/fsoc.2025.1591235)
Supplement: Supplementary file 1 [file Supplementary_file_1.pdf]

Supplementary Material

Causal Attributions of Poverty: A Social Stratification Analysis

Lionel Marquis, Ursina Kuh , Robin Tillmann

1 Question wording

Educational level: Low education (1): incomplete or completed compulsory school, 1 year school of commerce or domestic science; Upper Secondary (2): general training school, apprenticeship, high school; Vocational tertiary (3): vocational high school; (4) Teacher education, University of applied sciences, university academic high school, PhD.

Disposable household income*:* Disposable household income is provided by the Swiss Household Panel (SHP). The variable is computed at the basis of income amounts collected the individual questionnaires (and added at the household level), and on the household level (estimation of total income, transfers to other households). Costs for direct taxes and compulsory health care have been deducted using simulations. Disposable household income has been adjusted to the household size using the modified OECD scale, adjusted to inflation, and extreme values (top 1%) have been top coded to limit the impact of outliers.

Deprivation*:* Deprivation is asked with a list of 12 items. For each, household are first asked whether they have this item or not. Those who do not have an item, are then asked whether they do not have it due to financial reasons, which is counted as a deprivation. The final variable is dichotomous distinguishing household with at least two deprivations with the others. The deprivations considered are the following: One week holiday away from home per year, invitation of friends once a month, meal at restaurant once a month, car for private use, washing machine in home or for exclusive use, dishwasher, savings min. 500 CHF, 3rd pillar, go to the dentist when needed, fresh fruit and vegetables, complete meal every second day, own room.

Change of the financial situation*:* "Since (the last interview / or in the last 12 month) has your financial situation worsened or improved, if 0 means "very much worsened" and 10 "very much improved"?

Financial problems in childhood: "During your youth, did your family encounter serious financial problems?"

Perceived social class: "Do you feel you belong to a particular social class?" If the answer is yes: "Which social class do you have in mind?". If the answer is no: "If you have to choose, to which class would you say do you belong? To the (…)"

Answer categories: Working class, Lower middle class, Middle class, Upper middle class, Upper class, other class

Gender: Constructed variable (confirmation of previous information or registry information or grid information).

2 Principal component analysis of poverty attributions

A principal component analysis has been computed with the pooled data from the SHP (from years 2019-2021) following Halman and van Oorshot's (1999) method.

Figure A1: Principal component analysis for poverty attributions, factor loadings

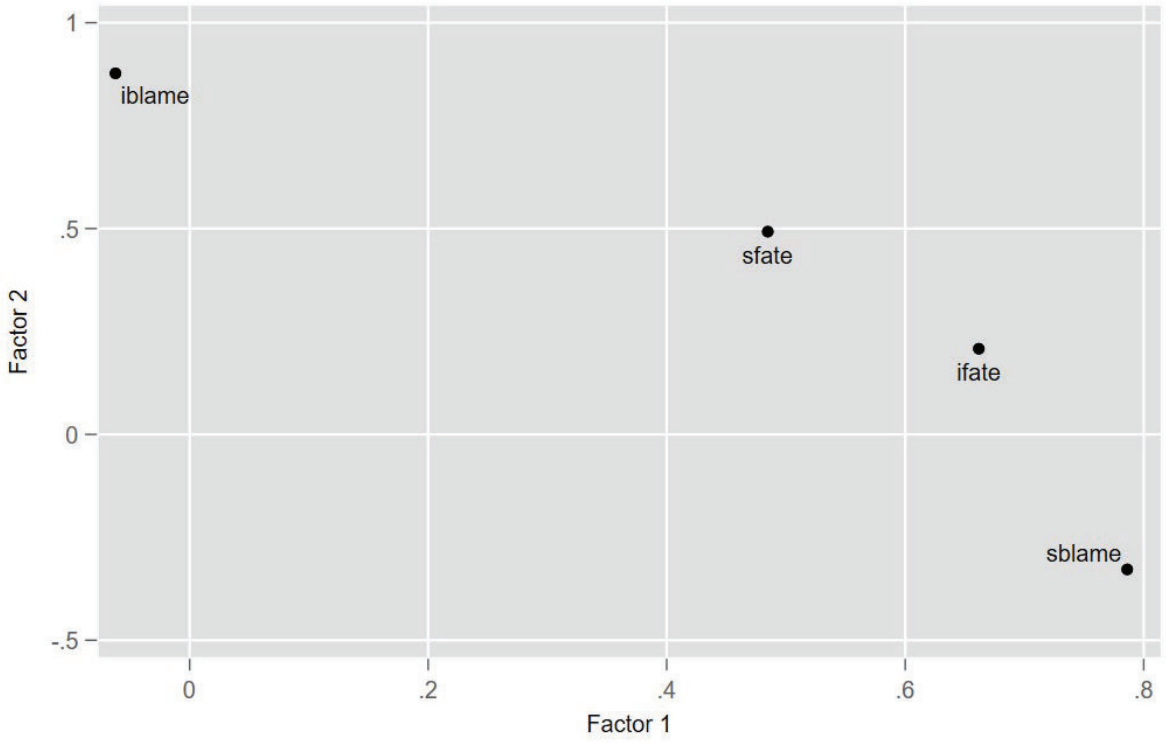

Source: SHP 2019-2021 (pooled data). Orthogonal varimax rotation with Kaiser normalized matrix.

3 Predicted values

Table A1: Financial resources model: predicted values for education and income quintiles

	IB	SB	IF	SF
Educational level				
Low	4.63 [4.53-4.73]	4.91 [4.81-5.01]	5.88 [5.79-5.97]	4.94[4.85-5.04]
Upper secondary	4.52 [4.47- 4.57]	5.01 [4.96-5.06]	5.98 [5.93-6.02]	4.83 [4.78-4.88]
Applied tertiary	4.35 [4.27- 4.44]	5.07 [4.98-5.15]	5.93 [5.86-6.01]	4.63 [4.55-4.72]
Academic tertiary	3.60 [3.53-3.67]	5.57 [5.50-5.64]	6.30 [6.23-6.37]	4.15 [4.08-4.23]
Income quintile:				
Income quintile:	4.33 [4.24-4.41]	6.09 [6.01-6.17]	5.05 [4.96-5.13]	4.77 [4.68-4.85]
2. Quintile	4.24 [4.17-4.31]	6.13 [6.07-6.19]	5.09 [5.02-5.16]	4.65 [4.58-4.72]
3 Quintile	4.26 [4.19-4.32]	6.11 [6.05-6.17]	5.09 [5.03-5.16]	4.68 [4.61-4.75]
4. Quintile	4.29 [4.22-4.36]	6.08 [6.02-6.14]	5.23 [5.16-5.29]	4.63 [4.56-4.70]
5. Quintile	4.33 [4.27-4.40]	5.81 [5.75-5.88]	5.22 [5.15-5.29]	4.55 [4.48-4.62]

Notes: Dependent variables: agreement with poverty attributions. Source: Swiss Household Panel (SHP).

Table A2: Income mobility model: predicted values change in financial situation

	IB	SB
Financial situation: strongly detoriated (0)	3.97 [3.83-4.11]	6.45 [6.32-5.57]
Unchanged	4.24 [4.20- 4.28]	6.09 [6.05-6.12]
Strongly improved	4.51 [4.39- 4.63]	5.72 [5.61-5.84]

Notes: Dependent variables: agreement with poverty attributions. Source: Swiss Household Panel (SHP).

Table A3: Social class model: predicted values for perceived class and work logic

	IB	SB
Perceived class		
None	4.40 [4.35-4.45]	5.79 [5.74 5.84]
Working class	4.28 [4.00 4.56]	6.22 [5.98 6.46]
Lower middle class	4.11 [3.92 4.31]	6.41 [6.24 6.58]
Middle class	4.24 [4.13 4.35]	6.10 [6.00 6.20]
Upper middle class	4.09 [3.95 4.23]	5.99 [5.86 6.12]
Class scheme		
**Not active**	4.16 [4.07 4.26]	6.00 [5.91 6.08]
**Large employers**	4.59 [4.20 4.99]	5.38 [4.94 5.81]
Self-employed professionals	3.78 [3.51 4.05]	5.87 [5.61 6.13]
**Small business owners with employees**	4.93 [4.66 5.20]	5.62 [5.37 5.87]
Small business owners without employees	4.42 [4.21 4.62]	5.73 [5.54 5.92]
Technical experts	4.11 [3.90 4.32]	6.08 [5.88 6.29]
Technicians	4.60 [4.37 4.84]	5.82 [5.61 6.03]
Skilled manual	5.11 [4.91 5.30]	5.73 [5.56 5.90]
Low-skilled manual	4.92 [4.49 5.35]	5.83 [5.47 6.20]
**Higher-grade managers and administrators**	4.34 [4.18 4.51]	5.63 [5.48 5.79]
**Lower-grade managers and administrators**	4.65 [4.42 4.87]	5.66 [5.45 5.87]
Skilled clerks	4.69 [4.53 4.85]	5.75 [5.61 5.89]
**Unskilled clerks**	3.79 [3.09 4.50]	6.62 [5.95 7.29]
**Socio-cultural professionals**	3.35 [3.15 3.55]	6.52 [6.33 6.72]
**Socio-cultural semi-professionals**	3.77 [3.62 3.93]	6.13 [5.99 6.27]
Skilled service	4.76 [4.57 4.96]	5.70 [5.52 5.88]
Low-skilled service	4.71 [4.48 4.95]	5.94 [5.73 6.16]

Notes: Dependent variables: agreement with poverty attributions. Source: Swiss Household Panel (SHP).

4 Wealth model

The SHP collected wealth data in 2016, 2020 and 2022 among its respondents. In 2020, the fresh sample (SHP IV), did not respond to the wealth question. Assuming relative stability of wealth over time in the short term, we have used previous and future wealth measures when available to impute missing values, as imputation is preferrable to excluding cases. Therefore, we use the measure from 2020 as a priority, the measure of 2016 as second priority and the measure of 2023 as third priority. Wealth measures are asked about a global estimation without distinction of different types of assets. The only exception is separate estimation of housing wealth for owner-occupiers. Missing values were imputed in case of non-response in the short term. It should be noted that due to the combination of significant share of non-response (about 25-30%) and imputed values, consideration of information of other years and the global measure, the estimates are not very precise. For that reason, we consider wealth in quintiles.

Table A4: Wealth model (OLS coefficients)

	IB		SB		IF		SF	
Educational level (Ref: low)								
Upper secondary	-0.111		0.060		0.108		-0.154	*
Applied tertiary	-0.278	**	0.038		0.166	*	-0.329	**
Academic tertiary	-1.051	**	0.434	**	0.637	**	-0.838	**
Income quintile: (Ref: 3rd)								
1. Quintile	-0.010		0.030		0.012		0.117	
2. Quintile	-0.040		0.035		-0.030		-0.043	
4. Quintile	0.015		-0.009		0.112	*	-0.050	
5. Quintile	0.044		-0.230	**	0.126	*	-0.198	**
Wealth quintile: (Ref: 3rd)								
1. Quintile	-0.053		0.341	**	-0.174	**		
2. Quintile	0.002		0.168	**	-0.088		-0.031	
4. Quintile	0.060		-0.053		-0.028		-0.036	
5. Quintile	0.124	*	-0.226	**	-0.042		0.011	
Age								
25-34	0.619	**	-0.148	*	0.399	**	0.121	
35-44	0.557	**	-0.098		0.562	**	0.121	
45-54	0.169	*	-0.183	**	0.466	**	0.120	
55-64	0.036		0.049		0.581	**	0.312	**
65-74	0.157	*	0.141	*	0.830	**	0.532	**
75 and more	0.364	**	0.046		1.009	**	0.979	**
swiss	-0.346	**	0.338	**	0.319	**	0.014	
male	0.699	**	-0.347	**	0.021		0.083	
cawi	-0.046		0.123	**	-0.292	**	0.018	
year/wave of data collection								
20	0.099	**	-0.073	**	0.191	**	-0.063	
21	0.123	**	0.166	**	0.527	**	0.127	**
Number of observations	31715		31807		31758		23932	
Adjusted R-squared	0.04		0.02		0.03		0.03	

Notes: Dependent variables: agreement with poverty attributions. ** : p<.01; *: p<.05. Source: Swiss Household Panel (SHP).

5 Additional social class models

Table A5: Parental social class model (OLS coefficients)

	IB		SB		IF		SF	
Occupational class position (Ref: inactive)								
Large employers	0.588	*	-0.191		-0.014		0.393	
Self-employed professionals	-0.394		0.044		0.497	*	0.018	
Small business owners with employees	0.637	**	-0.426	**	-0.078		-0.063	
Small business owners without employees	0.376	*	-0.095		0.113		0.197	
Technical experts	-0.263		-0.181		0.128		-0.290	
Technicians	0.211		-0.177		-0.404	*	-0.070	
Skilled manual	0.226		-0.406	**	-0.211		0.212	
Low-skilled manual	0.180		-0.866	**	-0.583		-0.191	
Higher-grade managers and administrators	-0.239		-0.154		0.409	**	-0.062	
Lower-grade managers and administrators	0.149		-0.704	**	0.190		0.135	
Skilled clerks	0.242		-0.164		-0.138		-0.064	
Unskilled clerks	0.041		0.799		-1.243	*	-0.352	
Socio-cultural professionals	-0.762	**	0.639	**	0.820	**	0.026	
Socio-cultural semi-professionals	-0.547	**	0.349	*	0.357	*	0.000	
Skilled service	0.380	*	-0.334	*	-0.188		0.110	
Low-skilled service	0.336		-0.076		-0.320		0.247	
Male	0.638	**	-0.344	**	0.076	*	0.034	
Age								
25-34	0.050		0.116		0.644	**	-0.269	**
35-44	0.035		-0.008		0.840	**	-0.180	*
45-54	-0.232	*	-0.192	*	0.703	**	-0.195	*
55-64	-0.301	**	-0.043		0.797	**	-0.022	
65-74	-0.134		0.040		0.982	**	0.323	**
75 and more	0.125		-0.022		1.147	**	0.811	**
swiss	-0.285	**	0.238	**	0.330	**	-0.054	
cawi	-0.050		0.133	**	-0.236	**	0.005	
Intercept	4.354	**	6.004	**	4.129	**	4.631	**
Number of observations	36547		36667		36580		35271	
Adjusted R-squared	0.02		0.01		0.02		0.01	

Notes: Dependent variables: agreement with poverty attributions. ** : p<.01; *: p<.05. Source: Swiss Household Panel (SHP).

Table A6: Stepwise class model: perceived social class (OLS coefficients)

	IB		SB	
Perceived social class (Ref: None)				
working class	0.002		0.430	**
lower middle class	-0.298	**	0.644	**
middle class	-0.237	**	0.347	**
upper middle class	-0.451	**	0.243	**
Low-skilled service	0.550	**	-0.051	
Age				
25-34	0.259	**	0.136	
35-44	0.100		0.009	
45-54	-0.162		-0.054	
55-64	-0.221	**	0.061	
65-74	-0.056		0.122	
75 and more	0.181		0.000	
Swiss nationality	-0.305	**	0.205	**
Men (Ref : women)	0.590	**	-0.356	**
Survey mode : cawi (Ref : CATI)	0.012		0.116	*
Intercept	4.436	**	5.706	
Number of observations	14100		14312	
R-squared	0.02		0.01	

Notes: Dependent variables: agreement with poverty attributions. ** : p<.01; *: p<.05. Source: Swiss Household Panel (SHP).

Table A7: Stepwise social class model: class scheme (OLS and FE coefficients)

	OLS				FE			
	IB		SB		IB		SB	
Occupational class position (Ref: inactive)								
Large employers	0.219		-0.597	**	0.354		0.220	
Self-employed professionals	-0.501	**	-0.165		-0.204		0.028	
Small business owners with employees	0.624	**	-0.323	**	0.198		-0.017	
Small business owners without employees	0.044		-0.133		-0.058		0.063	
Technical experts	-0.161		0.026		0.103		0.280	
Technicians	0.324	**	-0.190	*	0.095		0.157	
Skilled manual	0.857	**	-0.308	**	0.253		0.223	
Low-skilled manual	0.768	**	-0.180		0.735	**	0.242	
Higher-grade managers and administrators	0.074		-0.409	**	0.250		0.220	
Lower-grade managers and administrators	0.318	**	-0.377	**	0.051		0.032	
Skilled clerks	0.415	**	-0.300	**	0.163		0.110	
Unskilled clerks	-0.044		0.444		0.117		-0.440	
Socio-cultural professionals	-0.883	**	0.420	**	0.048		0.237	
Socio-cultural semi-professionals	-0.433	**	0.045		0.205		0.043	
Skilled service	0.586	**	-0.295	**	0.295	*	0.143	
Low-skilled service	0.370	**	-0.056		0.101		0.023	
Male	0.638	**	-0.344	**	0.076	*	0.034	
Age								
25-34	0.229	**	0.161	*	-0.180		-0.011	
35-44	0.181	*	0.118		-0.190		-0.050	
45-54	-0.135	*	-0.026		0.112		0.107	
55-64	-0.195	**	0.100		0.323		0.098	
65-74	0.047		0.081		0.532		0.104	
75 and more	0.337	**	-0.004		0.598		0.018	
swiss	-0.267	**	0.277	**	-0.507		0.242	
cawi	-0.016		0.149	**	-0.083		0.334	**
Intercept	4.156	**	5.938	**	4.428	**	5.627	**
Number of observations	34582		34702		35345		35469	
Adjusted R-squared	0.04		0.01		0.00		0.01	

Notes: Dependent variables: agreement with poverty attributions. ** : p<.01; *: p<.05. Source: Swiss Household Panel (SHP).

6 Controlling left-right position (OLS) coefficients

***Left-right scale**:* "When they talk about politics, people mention left and right. Personally, where do you position yourself, 0 means "left" and 10 "right"?"

Table A7: Financial resources model controlling for left-right position

	IB		SB		IF		SF	
Left-right	0.343	**	-0.260	**	-0.094	**	0.137	**
Educational level (Ref: low)								
Upper secondary	-0.164	**	0.098		0.058		-0.113	
Applied tertiary	-0.291	**	0.059		0.120		-0.317	**
Academic tertiary	-0.780	**	0.223	**	0.547	**	-0.674	**
Income quintile: (Ref: 3rd)								
1. Quintile	-0.037		0.033		-0.030		0.063	
2. Quintile	-0.032		0.072		-0.024		-0.047	
4. Quintile	0.014		-0.012		0.143	**	-0.067	
5. Quintile	-0.020		-0.208	**	0.142	**	-0.177	**
Deprivation (at least 2)	-0.159	**	0.411	**	0.038		0.056	
Gender : male (Ref : female)	0.535	**	-0.204	**	0.069		0.030	
Age								
25-34	0.306	**	0.176	*	0.337	**	-0.045	
35-44	0.182	*	0.168	*	0.575	**	0.004	
45-54	-0.250	**	0.052		0.464	**	-0.113	
55-64	-0.318	**	0.204	**	0.563	**	0.098	
65-74	-0.233	**	0.317	**	0.830	**	0.338	**
75 and more	-0.135		0.316	**	1.048	**	0.746	**
Nationality : Swiss (Ref : foreigner)	-0.247	**	0.280	**	0.451	**	-0.015	
year/wave of data collection								
20	0.110	**	-0.092	**	0.169	**	-0.043	
21	0.130	**	0.183	**	0.521	**	0.185	**
cawi	-0.152	**	0.191	**	-0.256	**	-0.013	
Intercept	2.995	**	6.749	**	4.214	**	4.131	**
Number of observations	31174		31272		31210		30878	
Adjusted R-squared	0.12		0.08		0.03		0.04	

Notes: Dependent variables: agreement with poverty attributions. ** : p<.01; *: p<.05. Source: Swiss Household Panel (SHP).

Table A8: Income mobility model controlling for left-right position

	IB		SB		IF		SF	
Left-right	0.339	**	-0.264	**	-0.093	**	0.138	**
Financial problem in youth	0.066	*	0.115	**	-0.062		0.002	
Change in financial situation	0.029	*	-0.048	**	0.004		-0.009	
Educational level (Ref: low)								
Upper secondary	-0.119		-0.002		0.112		-0.113	
Applied tertiary	-0.258	**	-0.061		0.188	*	-0.292	**
Academic tertiary	-0.776	**	0.083		0.585	**	-0.721	**
Age								
25-34	0.298	**	0.197	*	0.368	**	-0.017	
35-44	0.228	**	0.172	*	0.559	**	-0.024	
45-54	-0.221	**	0.035		0.474	**	-0.109	
55-64	-0.297	**	0.165	*	0.589	**	0.127	
65-74	-0.199	*	0.276	**	0.812	**	0.363	**
75 and more	-0.116		0.273	**	1.000	**	0.796	**
Swiss nationality	-0.144		0.249	**	0.482	**	0.008	
Men (Ref : women)	0.561	**	-0.179	**	0.054		0.013	
Survey mode : cawi (Ref : CATI)	-0.199	**	0.217	**	-0.321	**	-0.043	
year/wave of data collection								
20	0.056		-0.091	**	0.183	**	-0.066	
21	0.142	**	0.176	**	0.532	**	0.201	**
Number of observations	24943		25008		24974		24721	
Adjusted R-squared	0.12		0.07		0.03		0.04	

Notes: Dependent variables: agreement with poverty attributions. ** : p<.01; *: p<.05. Source: Swiss Household Panel (SHP).

Table A9: Social class model controlling for left-right position

	IB		SB		IF		SF	
Left-right	0.354	**	-0.247	**	-0.094	**	0.143	**
Perceived social class (Ref: None)								
working class	0.032		0.252		0.126		0.270	
lower middle class	-0.108		0.487	**	0.050		-0.043	
middle class	-0.024		0.217	**	0.376	**	-0.061	
upper middle class	-0.275	**	0.192	**	0.715	**	-0.120	
Occupational class position (Ref: inactive)								
Independent work logic								
Large employers	0.098		-0.359		-0.153		-0.515	*
Self-employed professionals	-0.266		-0.116		0.228		-0.407	*
Small business owners with employees	0.534	**	-0.123		0.025		-0.244	
Small business owners without employees	0.199		-0.162		-0.058		-0.233	
Technical work logic								
Technical experts	0.072		0.044		0.385	**	-0.639	**
Technicians	0.402	**	-0.144		-0.045		-0.421	**
Skilled manual	0.639	**	0.024		-0.305	*	0.038	
Low-skilled manual	0.353		0.156		-0.206		0.215	
Organisational work logic								
Higher-grade managers and administrators	0.069		-0.241	*	0.248	*	-0.419	**
Lower-grade managers and administrators	0.348	**	-0.190		0.149		-0.144	
Skilled clerks	0.388	**	-0.114		-0.072		-0.135	
Unskilled clerks	-0.435		0.631		-0.218		-0.235	
Interpersonal work logic								
Socio-cultural professionals	-0.473	**	0.326	**	0.425	**	-0.669	**
Socio-cultural semi-professionals	-0.164		0.042		0.039		-0.281	*
Skilled service	0.406	**	-0.188		-0.219		-0.001	
Low-skilled service	0.314	*	0.068		-0.169		0.021	
Age								
25-34								
35-44	0.232	*	0.238	*	0.429	**	-0.189	
45-54	-0.032		0.202	*	0.620	**	-0.055	
55-64	-0.410	**	0.179	*	0.534	**	-0.142	
65-74	-0.406	**	0.248	**	0.680	**	-0.055	
75 and more	-0.179		0.328	**	0.913	**	0.129	
Swiss nationality	-0.245	**	0.303	**	0.365	**	-0.006	
Men (Ref : women)	0.392	**	-0.221	**	0.074		0.034	
Survey mode : cawi (Ref : CATI)	-0.054		0.135	*	-0.162	*	-0.041	
Number of observations	12377		12426		12394		12267	
Adjusted R-squared	0.12		0.07		0.03		0.03	

Notes: Dependent variables: agreement with poverty attributions. ** : p<.01; *: p<.05. Source: Swiss Household Panel (SHP).
